# Effects of waste milk feeding on rumen fermentation and bacterial community of pre-weaned and post-weaned dairy calves

**DOI:** 10.3389/fmicb.2022.1063523

**Published:** 2023-01-16

**Authors:** Xinyue Zhang, Chuanteng Cheng, Jingyi Lv, Haixin Bai, Fang Sun, Chundong Liu, Chunlong Liu, Yonggen Zhang, Hangshu Xin

**Affiliations:** ^1^College of Animal Science and Technology, Northeast Agricultural University, Harbin, China; ^2^State Key Laboratory of Animal Nutrition, Beijing Engineering Technology Research Center of Raw Milk Quality and Safety Control, College of Animal Science and Technology, China Agricultural University, Beijing, China; ^3^Institute of Animal Husbandry, Heilongjiang Academy of Agricultural Sciences, Harbin, China; ^4^Harbin Wondersun the Cow Feeds the Reproduction Co., Ltd., Harbin, China; ^5^Northeast Institute of Geography and Agricultural Ecology, Chinese Academy of Sciences, Harbin, China

**Keywords:** rumen fermentation, calf, rumen bacteria, dietary, waste milk

## Abstract

The objective of this study was to investigate the effect of waste milk with antibiotic residue on rumen fermentation and rumen bacterial composition of dairy calves during pre-weaned and post-weaned periods. A total of 24 Holstein male calves (43.4 ± 0.93 kg body weight, mean ± standard error) were allocated into four blocks based on birth date. Dairy calves were supplied 100% milk replacer (MR, *n* = 8), 50% milk replacer mixed with 50% waste milk (MM, n = 8), or 100% waste milk (WM, n = 8). Ruminal samples were collected at 49 and 63 days of age and then subjected to determinations of pH value, volatile fatty acids (VFA), ammonia nitrogen (NH_3_–N) and 16S rRNA gene amplicon sequencing. The results showed that feeding WM had no effect on the pH value, the concentrations of VFA (acetic acid, propionic acid, butyric acid, isovaleric acid, valeric acid), and NH_3_–N in dairy calves compared to feeding MR. However, from 49 to 63 days of age, the pH value (*p* < 0.001) was significantly increased, while the levels of total VFA (*p* = 0.004), acetic acid (*p* = 0.01), propionic acid (*p* = 0.003) and valeric acid (*p* < 0.001) were significantly decreased. For rumen microorganisms, there was no differences in bacterial diversity among the treatments. But the relative abundance of *Veillonellaceae* was significantly lower (*p* = 0.05) in the calves fed WM than that from MR group at 49 days of age; however, no difference was detected at 63 days of age. Feeding WM to calves tended to reduce family *Veillonellaceae* and genus *Olsenella* in the rumen at 49 days of age (*p* = 0.049). Analysis of temporal changes in rumen bacteria based on alpha-diversity and beta-diversity as well as the microbial relative abundances did not exhibit any difference. In addition, relative abundances of *Clostridia_UCG-014*, *Prevotella*, *Syntrophococcus*, *Eubacterium_nodatum_group*, *Pseudoramibacter* and *Solobacterium* were correlated with rumen pH value and the concentrations of TVFA, propionic acid, isovaleric acid, valeric acid and NH_3_–N. In conclusion, compare to MR, calves supplied with WM had little changes on the rumen pH value, NH_3_–N or VFAs contents. Additionally, limited effects could be found on rumen microbiota in the calves fed WM. However, further studies needed to explore if there exist any long-term effects of early-life rumen microbiota modulation on dairy cows.

## 1. Introduction

The dairy farming industry occupies an essential part of the national economy and dairy calf producers must pay close attention to rearing costs and profit (Godden et al., 2005). Development and management during the calving period are directly associated with body structure and growth performance of mature cows and indirectly affect the economic returns of the dairy industry (Khan et al., 2016). A high-quality nutrient supply is crucial for calf growth and the whole milk is always considered the best liquid feed for calves (Moore et al., 2009). However, feeding whole milk to calves usually results in an economic loss to the producer and it is rarely adopted in dairy farms under normal circumstances all over the world (Britt et al., 2018). Thus, milk replacer containing protein, fat, vitamin, trace elements and immune factors is a common liquid feed for calves at present (Reid, 1956). From birth to weaning in detail, 300 ~ 400 kg of milk is consumed by each calf, which means that 10.52 yuan could be saved for per calf every day (raw milk price: 4.42 yuan/kg) after replacing raw milk with milk replacer (Si et al., 2015).

Besides milk replacer, an increase in waste milk is a manifestation of the continuous development and expansion of animal husbandry. Waste milk usually includes transitional milk, milk with antibiotic residues and other unsaleable milk (Zhang et al., 2022). Owing to the risk of antibiotic residues, waster milk is not suitable for human consumption. The annual production of waste milk has been reported to be 2 ~ 4% of the total milk production, which is equal to 0.8 ~ 1.6 million tons (Deng et al., 2017). Thus, the utilization of waste milk seems to be an effective method to minimize calf rearing costs. However, the controversy about waste milk with antibiotic residues on rumen fermentation and microorganism is ongoing. Vieira et al. (2021) reported higher acetate and propionate concentrations in the rumen of calves fed pasteurized waste milk rather raw milk. However, feeding milk replacer plus antibiotics (0.024 mg/l penicillin, 0.025 mg/l streptomycins, 0.1 mg/l tetracyclines, and 0.33 mg/l ceftiofur) to calves did not alter the content of TVFA in the rumen (Li et al., 2019). A richer rumen microbiota was exhibited in calves supplied waste milk than those fed raw milk (Zhang et al., 2019), whereas Owens and Basalan (2016) suggested that antibiotics could hinder rumen fermentation by inhibiting the microbial community. In previous study, we have evaluated the effects of waste milk on growth performance, immunity, and gut health of dairy calves (Zhang et al., 2022). Therefore, as another part of our systematic research, the present study was designed to investigate how the waste milk feeding affect rumen fermentation parameters and microbial community in dairy calves during the pre-weaning and post-weaning periods.

## 2. Materials and methods

The trial was conducted at Nestle Dairy Farm Institute (DFI, Harbin, Heilongjiang, China, E125°41′, N45°08′) from November 7, 2020 to February 2, 2021 and approved by the Ethical Committee of the College of Animal Science and Technology, Northeast Agriculture University (NEAU-[2011]-9). During the trial, medical treatments to disease were done according to the standard operating procedures at DFI.

### 2.1. Animals and treatments

The trial was performed according to a randomized complete block design. A total of 24 Holstein male calves were selected and assigned to four blocks according to date of birth and then randomly allocated to 1 of 3 groups: milk replacer (MR), mixed milk (50% milk replacer and 50% waste milk, MM), and pasteurized waste milk (WM). Taking feeding cost into consideration, milk replacer rather than raw milk was selected as the control liquid diet for calves in our study. The MR was obtained from the Land O′ Lakes company (Arden Hills, MN, United States), and the WM was obtained from the DFI, which included translation milk from cows on the second to third day after calving and antibiotic residue milk. The MR was diluted with warm water (46°C) at a ratio of 1:7, and the WM was pasteurized at 72°C for 15 s (Aust et al., 2013), then, both were cooled to 38 ~ 40°C for bottle feeding.

**Figure 1:**
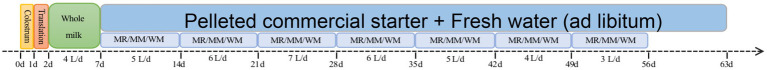
Feeding strategy.

According to the feeding regime in the farm, all calves were fed 4 l of colostrum within 3 h after birth and another 4 l of colostrum within 12 h to get enough passive transfer of immunity. Then, they were fed 4 l of translation milk at 2 days of age. From 3 to 7 days of age, 4 l of pasteurized whole milk was provided at 11:00 and 19:00 h. After that, the calves were bottled-fed at 07:30, 14:30, and 22:00 h with a step-up/step-down milk feeding procedure of 5, 6, 7, 6, 5, 4, and 3 l at week 2, 3, 4, 5, 6, 7, and 8 during the whole study, respectively (Figure 1). In addition, a pelleted commercial starter (Land O′ Lakes, Arden Hills, MN, United States) and fresh water were fed *ad libitum* from 8 days of age. All calves were weaned at 56 days of age, and the experiment was terminated at 63 days of age. The waste milk (50 ml), milk replacer (50 g) and starer samples (50 g) were collected every 16 days. The liquid samples were divided into two sample parts. The first fresh subsample was measured for antibiotic concentrations (Pony Testing International Group, Beijing, China) and the second milk subsample, milk replacer as well as starter samples were stored at –20°C and then determined for nutritional values (Tempini et al., 2018; Tables 1, 2).

**Table 1:** Nutrient levels of dietary treatment.

Items^1^	Diets^2^		
	MR	WM	Starter
DM (g/kg)	96.5 ± 0.08	–	88.4 ± 0.08
CP (g/kg DM)	3.1 ± 0.01	3.3 ± 0.01	21.7 ± 0.15
Fat (g/kg DM)	2.6 ± 0.006	4.4 ± 0.08	1.9 ± 0.07
Total solid (g/kg DM)	13.1 ± 0.02	12.8 ± 0.02	–
Lactose (g/kg DM)	6.7 ± 0.01	4.5 ± 0.01	–
Energy (MJ/kg)	19.4 ± 0.13	22.4 ± 0.09	–
NDF (g/kg DM)	–	–	68.3 ± 0.98
ADF (g/kg DM)	–	–	53.3 ± 0.86
Ash (g/kg DM)	–	–	0.2 ± 0.03

1 DM: dry matter; CP: crude protein; NDF: neutral detergent fiber; ADF: acid detergent fiber.

2 MR: 100% milk replacer; MM: 50% milk replacer mixed with 50% waste milk; WM: 100% waste milk.

**Table 2:** Varieties and concentrations of antibiotic in waste milk.

Date	Antibiotic category	Antibiotic content (μg/kg)
2020.11.23	Cefquinome	<4.0
	Ampicillin	1.4
2021.12.09	Lincomycin	<20.0
	Ampicillin	<1.0
2021.12.26	Lincomycin	<20.0
	Ampicillin	<1.0
	Benzylpenicillin	<2.0
2021.01.12	Lincomycin	203.0
	Ampicillin	<1.0
	Benzylpenicillin	85.2

### 2.2. Sampling and analysis

#### 2.2.1. Ruminal fluid collection and measurement

Ruminal fluid was collected by an oral stomach tube after 3 h of morning feeding from the calves at 49 and 63 days of age in all groups. The initial 100 ml of rumen fluid was discarded to avoid saliva contamination. Then, the subsequent rumen fluid (100 ml) was extracted and filtered through four layers of cheesecloth, and the pH value was measured immediately with a pH electrode (Sartorius, Göttingen, Germany) after collection. After that, the filtered liquid was separated into two sections: one section was directly placed in liquid nitrogen and then transferred to –80°C for 16S rRNA sequencing analysis; the other was stored at –20°C for VFA and NH_3_–N analyses. Specifically, 1 ml metaphosphoric acid (25%, *w*/*v*) was added to a 5 ml sample for VFA analysis, and 0.1 ml vitriol (50%, *v*/*v*) was added to a 5 ml sample to remove the albumen precipitate for NH_3_–N analysis.

Volatile fatty acids concentrations were measured by a gas chromatography (GC-2010 Gas Chromatograph, Shimadzu Corporation) with a gas chromatography column (Agilent J&W HP-INNOWax) with the length and diameter were 30 cm and 0.25 mm, respectively. Before quantification, rumen fluid was centrifuged at 10000 r/min for 15 min; and then the supernatant was detected after filtering through a water membrane filter. The concentration of NH_3_-N was detected using a spectrophotometer (WFZ UV-2000, Unico Instrument Co., Ltd., Shanghai, China) according to the phenol-sodium hypochlorite colorimetry method described by Broderick and Kang (1980).

#### 2.2.2. 16S rRNA gene sequencing

Approximately 1.5 ml rumen fluid of each sample was delivered to Novogene company, and the specific amount for measurement was around 0.5–1.0 ml. The DNA extraction, reverse transcription, and PCR amplification of rumen fluid samples were performed by Novogene Company (Tianjin, China). The bacterial community was profiled by sequencing the V3-V4 hypervariable region of 16S rRNA gene using the primers 314\u00B0F (5′-CCTAYGGGRBGCASCAG-3′) and 806 R (5′-GGACTACNNGGGTATATAAT-3′; Henderson et al., 2015), and a 6-bp barcode sequence was added to the 5 ‘end of each upstream and downstream primer to distinguish different samples. The amplicons were sequenced (2 × 250 bp) by PE250 sequencing using the Novaseq 6000 platform, and then the raw data were acquired for analysis.

Analysis of raw 16S rRNA gene sequence data was performed using the Quantitative Insights into Microbial Ecology 2 (Bolyen et al., 2019; QIIME2, version 2021.04). The DADA2 workflow was used to remove barcodes, primers, and low-quality and undetected sequences and to merge the paired end reads as described by Xin et al. (2021). Sequences with quality control scores lower than 30% were manually removed to obtain high-quality clean tags. Taxonomic classification was performed for each operational taxonomic unit (OTU) based on 99% sequence similarity according to the SILVA database (SILVA Release 138). Only bacterial taxa with a relative abundance > 0.1% in at least four calves (half of sample size) in each group were considered as identified in this study. Moreover, subsequent analysis of alpha and beta diversity and determination of differences in bacterial abundance was performed based on the data output. All sequences were deposited in the NCBI Sequence Read Archive (accession number: SRR21731694 to SRR21731762).

### 2.3. Statistical analysis

Ruminal fermentation indicator analyses were performed using the PROC MIXED procedure of Statistical Analysis System 9.4 (SAS Institute Inc., Cary, NC, United States). A randomized complete block design was used in this study, where the effects of treatment and age and the interaction of treatment and age were determined according to the following model:


$$Y_{ijkl}=\mu +A_{i}+T_{j}+AT_{ij}+B_{k}+\varepsilon_{ijkl}$$

where *Y_ijkl_* is the dependent variable, *μ* is the overall mean, *A_i_* is the fixed effect of treatment, *T_j_* is the effect of age, *AT_ij_* is the interaction effect of age and treatment, *B_k_* is the block effect, and *ε*_*ijkl*_ is the random residual error. The results are presented as the least significant difference (LSD).

The stacked bar chart was generated at different levels using R software (version 4.0.2; R Foundation for Statistical Computing, Vienna, Austria). The difference in rumen bacterial abundance was assessed using the Kruskal–Wallis test in R, and the *p* value was adjusted based on the false discovery rate (FDR) according to the Benjamini–Hochberg algorithm. Effects on alpha diversity and principal coordinate analysis (PCoA) were tested in QIIME2 (version 2021.04), and the comparison among treatments was analyzed using the Kruskal–Wallis test and permutational multivariate analysis of variance (PERMANOVA), respectively. The *p* value was adjusted based on the FDR. Spearman’s rank correlation was used to identify the relationship between the rumen bacteria and rumen fermentation profiles (VFA, NH_3_–N, and pH value) using the “corrplot” package in R. Statistical significance was declared at *p* ≤ 0.05, and trends were declared at 0.05 < *p* ≤ 0.10. Since one calf died during the trial; thus, the corresponding data were excluded from the dataset before analysis.

## 3. Results

### 3.1. Effects of waste milk feeding on rumen fermentation parameters in dairy calves

**Figure 2:**
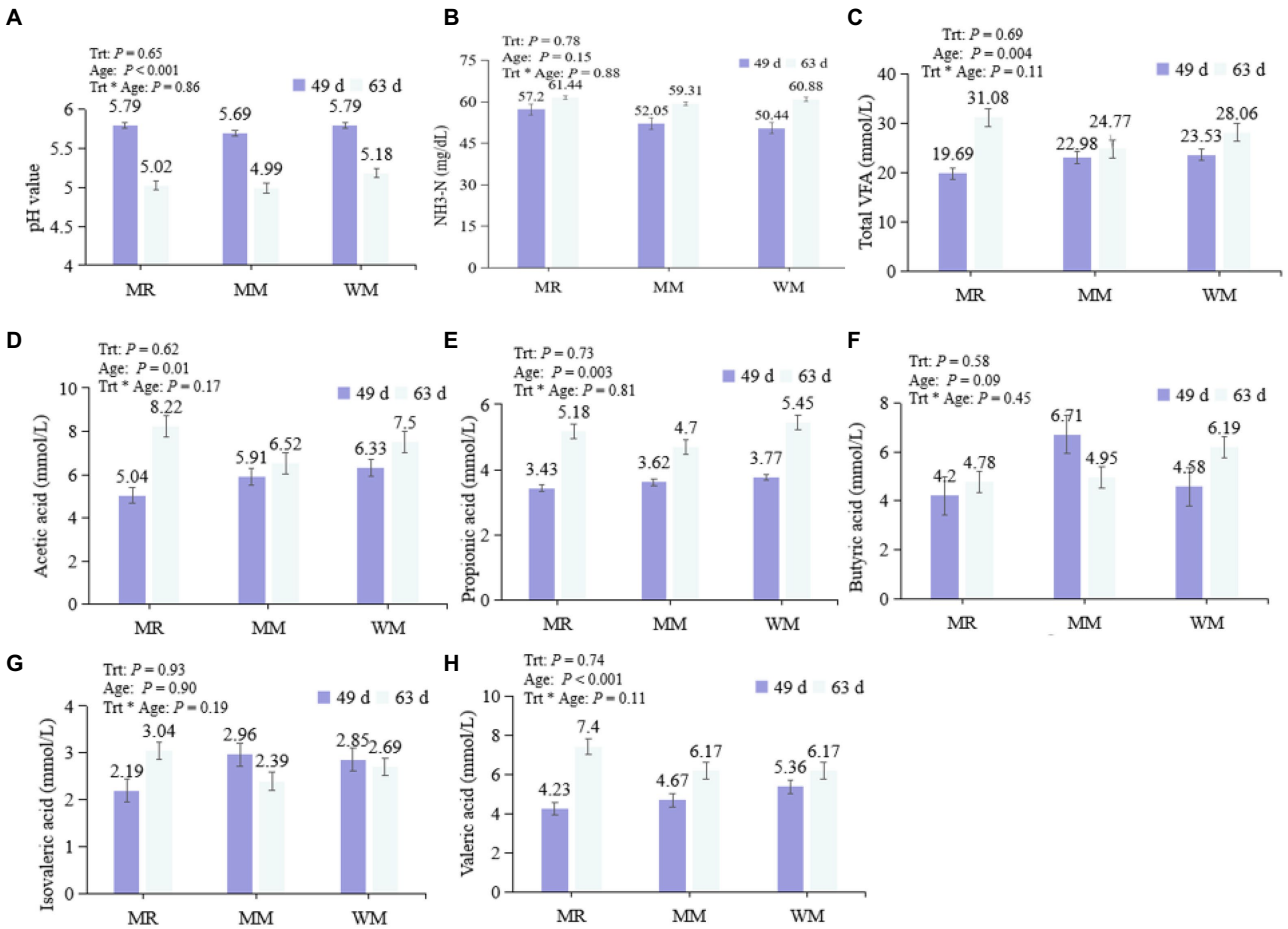
Effect of waste milk feeding on rumen pH value **(A)**, NH_3_-N **(B)** and volatile fatty acids **(C–H)** in dairy calves.

As shown in Figure 2, no interactive effect was found between treatment and age, and no significant difference was observed in the pH value, total VFA, acetic acid, propionic acid, butyric acid, isovaleric acid, valeric acid, and NH_3_–N concentrations among the treatments during pre-weaned or post-weaned periods. And the rumen pH value of calves in the pre-weaned period was significantly higher than that in the post-weaned period; while concentrations of total VFA, acetic acid, propionic acid and valeric acid exhibited the opposite trend.

### 3.2. Effects of waste milk feeding on rumen bacterial diversity in dairy calves

**Figure 3:**
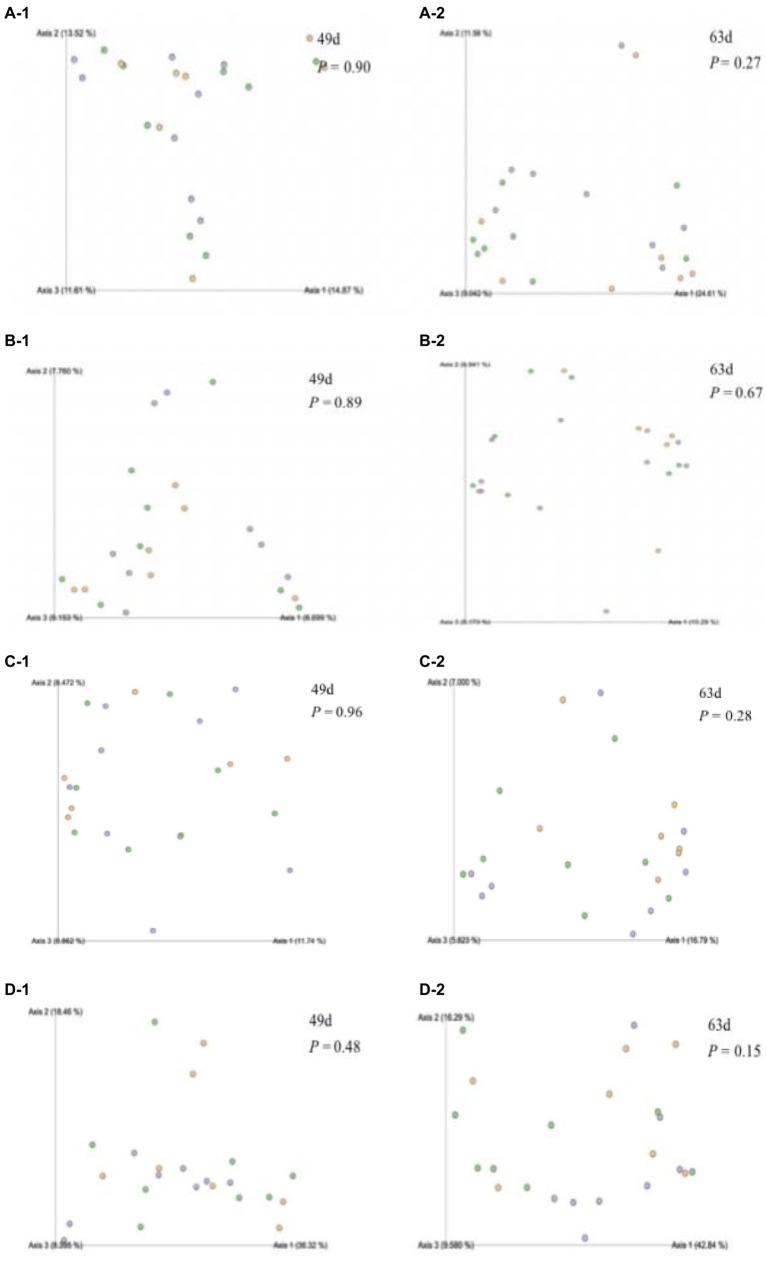
Effect of waste milk feeding on rumen bacterial beta-diversity in dairy calves **(A-1)** Bray-distance matrix values at 49 d of age; **(A-2)** Bray-distance matrix values at 63 d of age of calves; **(B-1)** Jaccard-distance matrix values at 49 d of age; **(B-2)** Jaccard-distance matrix values at 63 d of age of calves; **(C-1)** Unweighted-distance matrix values at 49 d of age; **(C-2)** Unweighted-distance matrix values at 63 d of age of calves; **(D-1)** Weighted-distance matrix values at 49 d of age and **(D-2)** Weighted-distance matrix values at 63 d of age of calves.

**Figure 4:**
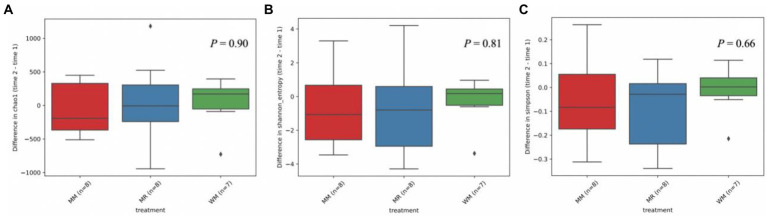
Differential abundance of alpha diversity based on Chao1 **(A)**, Shannon **(B)** and Simpson **(C)** index in dairy calves between 49 and 63 days of age.

**Figure 5:**
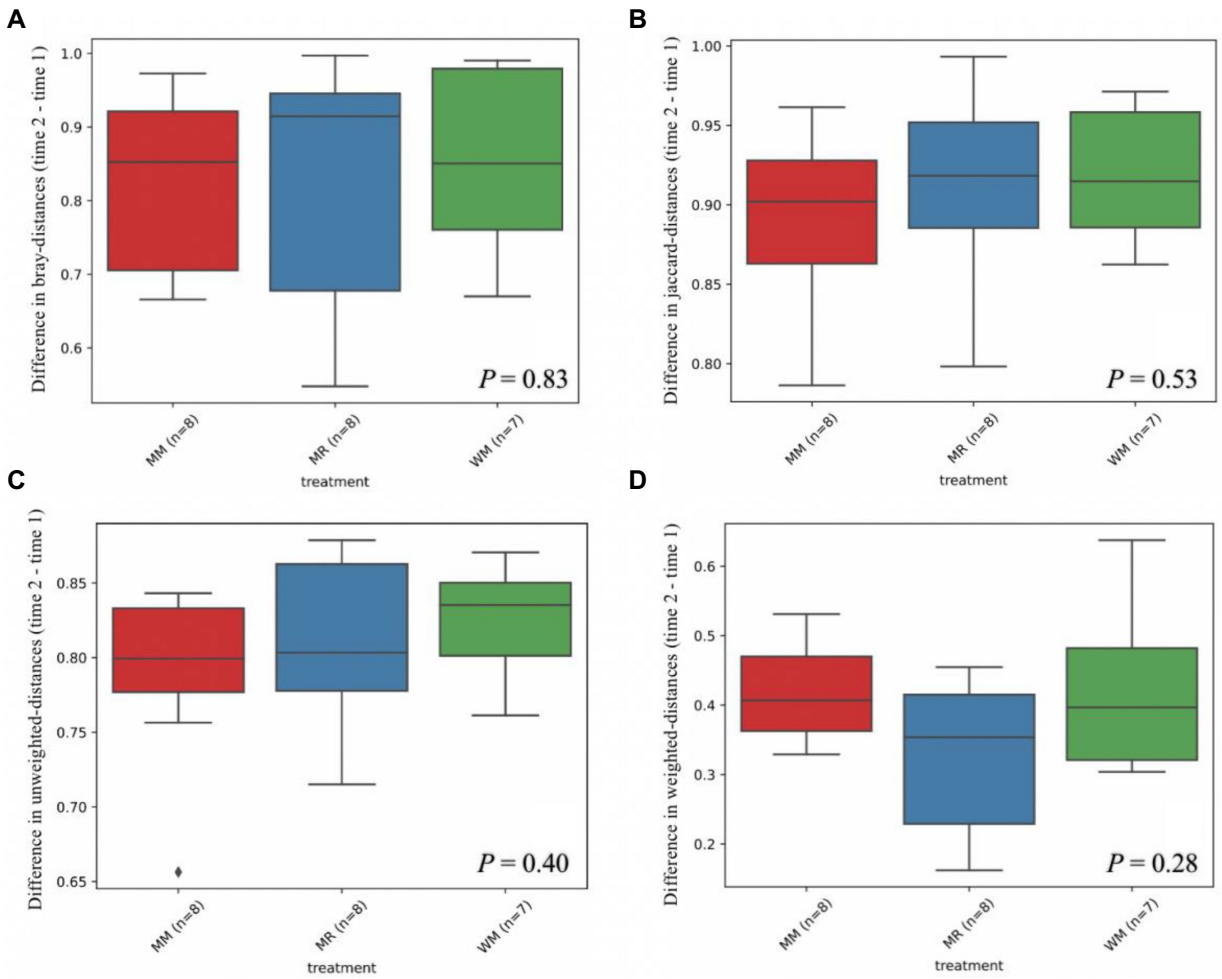
Differential abundance of beta diversity based on Bray-distance **(A)**, Jaccard-distance **(B)**, Unweighted-distance **(C)** and Weighted-distance **(D)** in dairy calves between 49 and 63 days of age.

The rumen bacterial diversity data are shown in Table 3 and Figure 3. No matter at 49 days of age or at 63 days of age, all the diversity parameters were not altered in dairy calves treated by different feeding strategies. In addition, the temporal changes in alpha-diversity and beta-diversity of the rumen microbiota were not significant among the three groups (Figures 4, 5). Among them, the ace indices (49 days: MR vs. MM vs. WM = 759: 652: 687; 63 days: MR vs. MM vs. WM = 768: 564: 674); the chao indices (49 days: MR vs. MM vs. WM = 600: 770: 722; 63 days: MR vs. MM vs. WM = 762: 560: 670); the Shannon indices (49 days: MR vs. MM vs. WM = 5.3: 6.3: 5.7; 63 days: MR vs. MM vs. WM = 5.5: 4.8: 5.2); the Simpson indices (49 days: MR vs. MM vs. WM = 0.9: 1.0: 0.9; 63 days: MR vs. MM vs. WM = 0.85: 0.84: 0.90).

**Table 3:** Effects of waste milk feeding on rumen bacterial alpha diversity in dairy calves.

Items	Age (days)	Diets^1^			SEM	*p*-Value
		MR	MM	WM		
OTU	49	713	619	637	48.76	0.74
	63	756	549	664	74.08	0.73
Ace	49	759	652	687	52.08	0.90
	63	768	564	674	75.09	0.73
Chao	49	600	770	722	51.78	0.46
	63	762	560	670	74.40	0.73
Shannon	49	5.3	6.3	5.7	0.26	0.26
	63	5.5	4.8	5.2	0.36	0.81
Simpson	49	0.9	1.0	0.9	0.02	0.10
	63	0.85	0.84	0.90	0.02	0.79

1 MR: 100% milk replacer; MM: 50% milk replacer mixed with 50% waste milk; WM: 100% waste milk.

### 3.3. Effects of waste milk feeding on relative microbial abundance in dairy calves

In total, 10 bacterial phyla, 39 bacterial families, 60 bacterial genera and 7 bacterial species were identified in the rumen of calves at 49 days of age. And the microbiota at the phylum, family, genus and species levels were 7, 26, 43, and 3, respectively, for the calves at 63 days of age.

**Figure 6:**
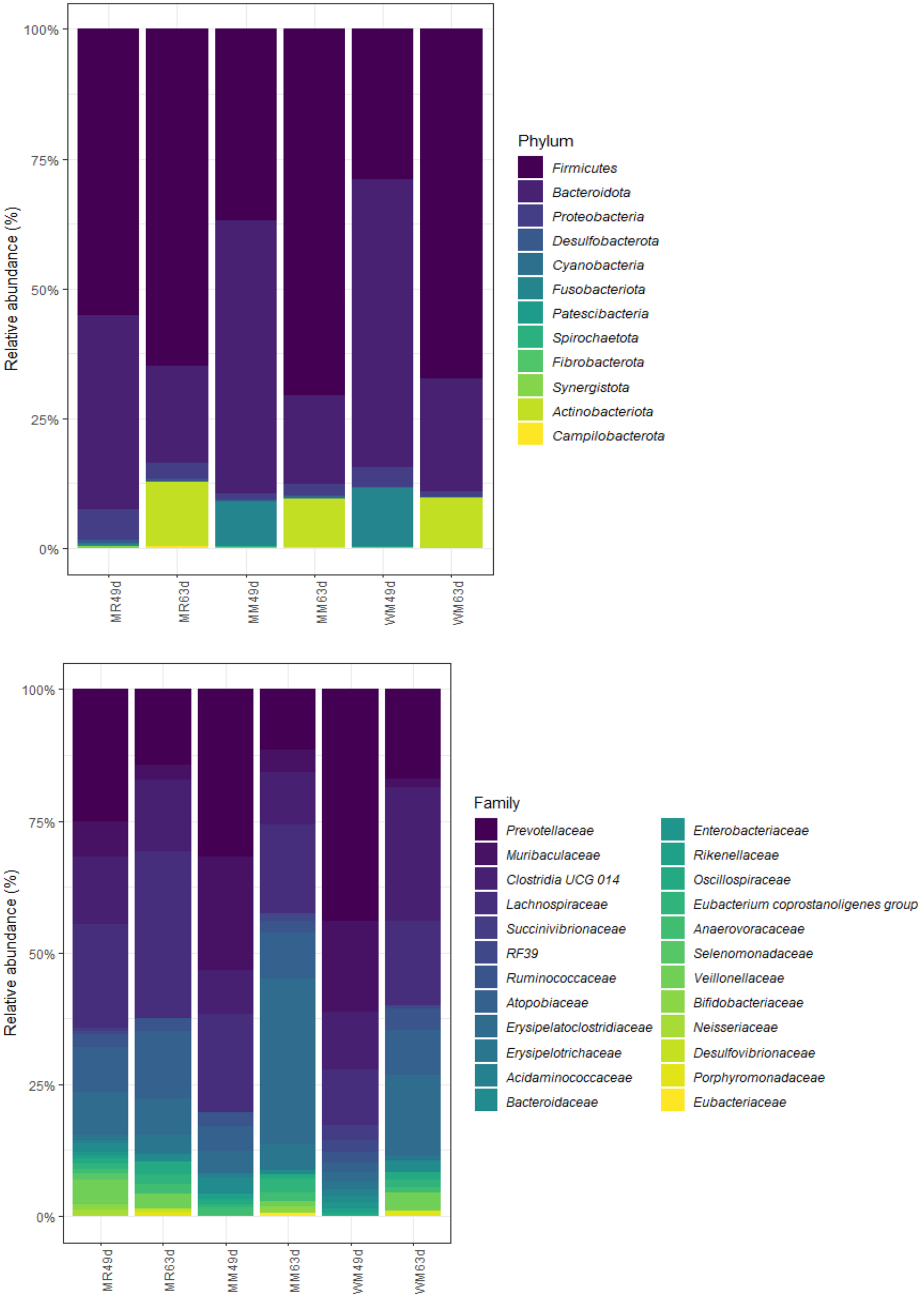
Effects of waste milk feeding on rumen bacterial phylum and family (relative abundance > 0.5%) community compositions in dairy calves.

**Figure 7:**
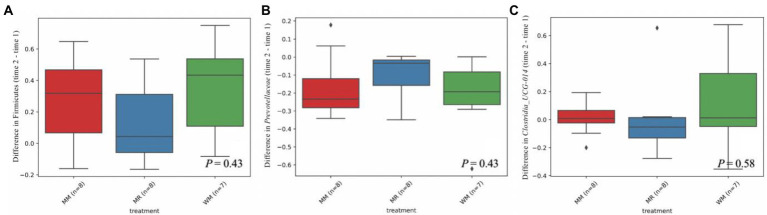
Differential abundance of Firmicutes **(A)**, *Prevotellaceae* **(B)** and *Clostridia_UCG-014* **(C)** in dairy calves between 49 and 63 days of age.

As shown in Tables 4, 5 and Figure 6, Firmicutes and Bacteroidetes were the predominant phyla in calves in both pre-weaned and post-weaned periods from different groups. We compared all bacterial relative abundances at different levels among all treatments and found that only the relative abundance of *Veillonellaceae* in the WM group (0.3%) was significantly lower than that in the MR group (4.2%) at 49 days of age; however, no difference was detected at 63 days of age. Moreover, the temporal change in the rumen top microbiota in calves between 49 and 63 days of age did not exhibit any differences (Figure 7).

**Table 4:** Effects of waste milk on rumen bacterial community compositions in dairy calves at 49 days of age(%).

Phylum level	Family level	Genus level	MR	MM	WM	SEM	*p*-Value
Bacteroidota			33.2	49.5	53.8	11.2	0.28
	Prevotellaceae		21.8	25.9	36.4	3.4	0.22
		Prevotella	16.8	17.8	28.9	3.0	0.15
	Muribaculaceae		5.8	17.6	14.2	3.3	0.12
		Muribaculaceae	5.8	17.6	14.2	3.3	0.12
Firmicutes			49.0	34.6	28.1	5.9	0.22
	Veillonellaceae		4.2^a^	0.3^b^	0.3^b^	1.0	0.05
	Clostridia UCG 014		11.2	6.9	9.2	2.5	0.30
		Clostridia_UCG-014	11.2	6.9	9.2	2.5	0.30
		RF39	0.5	0.3	1.9	0.5	0.62
	Lachnospiraceae		17.1	15.3	8.7	3.6	0.36
	Erysipelatoclostridiaceae		7.1	3.5	1.5	1.8	0.54
Fusobacteriota			0.4	8.0	10.8	2.3	0.94
Proteobacteria			5.2	1.1	3.7	0.8	0.21
Cyanobacteria			0.1	0.2	0.2	0.03	0.54
Actinobacteriota	Atopobiaceae	Olsenella	7.5	3.8	1.6	1.0	0.06

Only significant and top five rumen bacterial community composition of every level in calves at 49 days are included.

**Table 5:** Effects of waste milk feeding on rumen bacterial community compositions in dairy calves at 63 days of age(%).

Phylum level	Family level	Genus level	MR	MM	WM	SEM	*p*-Value
Firmicutes			62.5	60.5	62.6	3.2	0.97
	Clostridia UCG 014		11.4	8.0	22.4	4.1	0.59
		Clostridia_UCG-014	11.4	8.0	22.4	4.1	0.59
	Lachnospiraceae		26.8	13.5	14.2	2.9	0.10
		Shuttleworthia	0.5	0.2	3.2	0.8	0.32
	Erysipelatoclostridiaceae		6.0	25.2	13.4	3.9	0.26
		Erysipelotrichaceae_ UCG-002	5.8	25.1	13.2	3.9	0.15
Bacteroidota			18.0	14.6	20.2	3.2	0.52
	Prevotellaceae		12.0	9.3	15.0	2.2	0.33
		Prevotella	10.0	5.4	13.4	1.7	0.19
Actinobacteriota			12.0	8.0	9.0	2.5	0.86
	Atopobiaceae		10.7	7.1	7.7	2.5	0.83
		Olsenella	10.7	7.1	7.7	2.5	0.83
Proteobacteria			3.0	2.0	0.8	0.8	0.77
Desulfobacterota			0.5	0.2	0.1	0.1	0.37

Only top five rumen bacterial community composition of every level in calves at 63 days are included.

### 3.4. The correlations between rumen bacterial abundances and fermentation indices

As shown in Table 6, Spearman’s rank correlation was used to identify the relationship among the different rumen bacteria and ruminal fermentation indices (pH, TVFA, propionic acid, isovaleric acid, valeric acid, and NH_3_–N). The rumen pH value was negatively correlated with the relative abundances of *Erysipelatoclostridium* (*r* = 0.41, *p* = 0.05), *Eubacteriaceae* (*r* = 0.53, *p* = 0.01), and *Pseudoramibacter* (*r* = 0.50, *p* = 0.01). TVFA content was negatively correlated with the relative abundance of *Syntrophococcus* (*r* = 0.51, *p* = 0.01). Propionic acid concentration was positively correlated with the relative abundance of *Prevotella* (*r* = 0.44, *p* = 0.03). Isovaleric acid content was negatively correlated with the relative abundances of *Clostridia_UCG-014* (*r* = 0.45, *p* = 0.03) and *Syntrophococcus* (*r* = 0.51, *p* = 0.01). Valeric acid content was negatively correlated with the relative abundances of *Syntrophococcus* (*r* = 0.48, *p* = 0.02). NH_3_–N content was negatively correlated with the relative abundances of *Cyanobacteria* (*r* = 0.43, *p* = 0.04), *Eubacteriaceae* (*r* = 0.43, *p* = 0.04), *Eubacterium nodatum group* (*r* = 0.48, *p* = 0.02), *Pseudoramibacter* (*r* = 0.44, *p* = 0.04), and *Solobacterium* (*r* = 0.47, *p* = 0.02).

**Table 6:** The correlations between rumen bacteria and fermentation parameters.

	pH	TVFA	Propionic acid	Isovaleric acid	Valeric acid	NH_3_–N
Phylum						
*Cyanobacteria*						−0.43 0.04
Family						
*Erysipelatoclostridiace*	−0.41 0.05					
*Clostridia_UCG-014*				−0.45 0.03		
*Eubacteriaceae*	−0.53 0.01					−0.43 0.04
Genus						
*Prevotella*			0.44 0.03			
*Syntrophococcus*		−0.51 0.01		−0.51 0.01	−0.48 0.02	
*Eubacterium_nodatum_group*						−0.48 0.02
*Pseudoramibacter*	−0.50 0.01					−0.44 0.04
*Solobacterium*						−0.47 0.02

## 4. Discussion

### 4.1. Ruminal fermentation profiles

The rumen pH value and VFA and NH_3_–N contents are essential indicators of rumen function and homeostasis. The pH value is affected by various factors, including the concentrations of VFA, NH_3_–N and saliva (Li et al., 2019). In our study, the pH value of rumen liquid was unchanged in different treatments at the ages of 49 and 63 days. However, we found that the rumen pH value in pre-weaned calves was significantly higher than that in post-weaned calves, indicating the starter intake enhanced rumen development as the calves aged. As a result, the VFAs accumulated and pH values decreased as the nutrients in the starter were fermented by the rumen microorganisms (Robinson et al., 1987; Maynou et al., 2019). Mutually, the pH decreases also promoted the VFAs absorption *via* rumen epithelial cells, further facilitating the rumen development of calves (Baldwin and McLeod, 2000).

Short-chain fatty acids are the main source of energy for calves, and they are also considered as essential substances for the development of rumen epithelial cells (den Besten et al., 2013). In this study, there was no significant difference in rumen TVFA concentration among the three groups, which was consistent with the results of a previous study (Li et al., 2019) in which quantitative antibiotics (including 0.024 mg/l penicillin, 0.025 mg/l streptomycin, 0.1 mg/l tetracycline, and 0.33 mg/l ceftiofur) were added to milk replacers. Milk replacer combined with antibiotics increased the concentration of acetic acid in their research; but no significant change was detected when the calves were fed waste milk in our trial. The discrepancies might be due to the various types and concentrations of antibiotics used these two studies. The antibiotic residue contents included in the waste milk used in our study (Table 2) might not be sufficient to influence the population of microorganisms dominating the production of acetic acid. However, further studies would be needed to explore. Additionally, we found that acetic acid, propionic acid, valeric acid and TVFA concentrations were significantly greater at 63 days of age than at 49 days of age, and butyric acid content also tended to increase with time, which was consistent with Kong et al. (2019) and Hao et al. (2021).

NH_3_–N is crucial for microbial protein synthesis, and the NH_3_–N concentration in this trial was not different among the groups. This might be related to the similar protein intake which was observed in our previous report (Zhang et al., 2022). In the present study, the NH_3_–N contents in calves from different treatments were all within the recommendation described by Wanapat and Pimpa (1999), indicating that the NH_3_–N provision might be sufficient to meet the calves’ metabolic requirements.

### 4.2. Ruminal bacterial diversity and taxonomic compositions

The growth development of calves is largely related to rumen microbial communities (Malmuthuge and Guan, 2017). Although in traditional theory system, liquid milk is regarded to bypass the rumen and directly access the abomasum *via* closed esophageal groove in dairy calves; the rumen microbiota colonization and distribution would be affected by waste milk with antibiotics owing to the interlinked gastrointestinal system (Li et al., 2017). Owens and Basalan (2016) believed that antibiotics could hinder rumen fermentation by inhibiting the microbial community. However, our data showed that there were no differences in microbial diversity. This implied that the rumen microbiome composing a sophisticated network of symbiosis is a very complex system; and the types and doses of antibiotics have inconsistent effects on microbial diversity. It has been reported that *Veillonellaceae* can contribute to some inflammatory diseases, like primary sclerosing cholangitis (Gevers et al., 2014), implying that feeding waste milk to calves might have a potential to avoid inflammation occurrence, according to the lower abundance of calves in WM group. However, no difference in rumen bacterial taxonomic compositions was observed in calves at 63 days of age. As calves aged, the rumen microbiota tended to be stable after weaning compared to the pre-weaned period (Klein-Jöbstl et al., 2014). On the other hand, the antibiotic residues in WM may not have a long-term effect on rumen microbiota (Van Vleck Pereira et al., 2016). Therefore, it is essential and necessary to track and monitor the profiles of antibiotic resistance genes in the rumen when calves are supplied with WM. The lack of any changes in differential abundance in alpha-diversity indices or PCoA distance of rumen bacteria among treatments suggested that rumen bacterial community could not be distinguished in pre-weaned and post-weaned calves. It should be noted that the waste milk used in our study contained various types of antibiotics with different dosages, which made the effects on microbial flora were complicated. So further studies are needed to deeply explore the influence of each kind of antibiotic residue in the waste milk fed to dairy calves.

### 4.3. Correlations of rumen bacterial abundances with fermentation indicators

As well known, changes in pH, VFA and NH_3_–N are highly related to changes in microbiota (Kong et al., 2022). Different bacteria play different roles in the rumen. In our study, *Prevotella* which was the dominant bacteria being responsible for starch fermentation to produce propionate in the rumen (Strobel, 1992), was positively correlated with propionic acid content. *Pseudobutyrivibrio* can digest and degrade some complex plant polysaccharides and then produce VFA for use by ruminants (Palevich et al., 2020). It is easy to understand that the relative abundance of *Pseudobutyrivibrio* was negatively correlated with the pH value observed in our study.

## 5. Conclusion

In conclusion, WM with antibiotic residue could not change the rumen pH value and the concentration of NH_3_–N and VFAs. And the rumen bacterial diversity and relative abundance were also not altered by different liquid diets. In addition, in-depth studies with large sample size are needed to explore if there exist any long-term effects of early-life rumen microbiota modulation on dairy cows.

## Data availability statement

The original contributions presented in the study are included in the article/supplementary material, further inquiries can be directed to the corresponding authors.

## Ethics statement

The animal study was reviewed and approved by Ethical Committee of the College of Animal Science and Technology, Northeast Agriculture University. Written informed consent was obtained from the owners for the participation of their animals in this study.

## Author contributions

XZ and CC: methodology, formal analysis, investigation, writing-original draft, and visualization. JL and HB: investigation and formal analysis. FS: methodology. CL: investigation and resources. CL and YZ: review. XH: conceptualization, methodology, resources, and writing-review and editing, visualization, supervision, and funding acquisition. All authors contributed to the article and approved the submitted version.

## Funding

The work was supported by the earmarked fund for CARS-36 and Heilongjiang Provincial Dairy Industry and Technology System.

## Conflict of interest

CL was employed by Harbin Wondersun the Cow Feeds the Reproduction Co., Ltd.

The remaining authors declare that the research was conducted in the absence of any commercial or financial relationships that could be construed as a potential conflict of interest.

## Publisher’s note

All claims expressed in this article are solely those of the authors and do not necessarily represent those of their affiliated organizations, or those of the publisher, the editors and the reviewers. Any product that may be evaluated in this article, or claim that may be made by its manufacturer, is not guaranteed or endorsed by the publisher.
